# True Generalized Microdontia and Hypodontia with Spondyloepiphyseal Dysplasia

**DOI:** 10.1155/2013/685781

**Published:** 2013-12-12

**Authors:** Anita Singhal, Parul Singhal, Ranjan Gupta, Kush Dev Jarial

**Affiliations:** ^1^Department of Oral Pathology and Microbiology, H.P. Government Dental College and Hospital, Shimla, Himachal Pradesh 171001, India; ^2^Department of Pedodontics and Preventive Dentistry, H.P. Government Dental College and Hospital, Shimla, Himachal Pradesh 171001, India; ^3^Department of Prosthodontics, H.P. Government Dental College and Hospital, Shimla, Himachal Pradesh 171001, India; ^4^Department of Pediatrics, Indira Gandhi Medical College & Hospital, Shimla, Himachal Pradesh 171001, India

## Abstract

Spondyloepiphyseal dysplasia (SED) is a descriptive term used for group of inherited disorders of bone growth resulting in short stature, skeletal abnormalities, and problems with hearing and vision. SED have three major forms, SED congenital, pseudoachondroplastic SED, and SED tarda. SED tarda is milder than SED congenita. True generalized microdontia is a rare condition in which all the teeth are abnormally small. This is a report of a rare case having SED with generalized microdontia in a 26-year-old patient.

## 1. Introduction 

SED refers to a heterogeneous group of disorders with primary involvement of vertebrae and epiphyseal centers of long bones. Three major types of SED are recognized SED congenital, pseudoachondroplastic SED, and SED tarda. Spondyloepiphyseal dysplasia (SED) was first described in 1966 by Spranger and Wiedemann and is now also known as Wiedemann-Spranger syndrome [1]. SED congenita is inherited as an autosomal dominant condition and is associated with a highly disproportional reduction in growth and severe coxa vara, and the milder tarda form which is an X-linked recessive condition, in which growth in adolescence is defective after normal childhood development. Clinically, SED is characterized by short stature (120 to 140 cm), often significant lordosis, pectus carinatum and may have associated features of myopia, retinal detachment, deafness, cleft lip cleft palate, and muscular hypotonia [2]. The genetic modes for SED tarda are various. Initially heredity was described as X-linked, but in later reports both autosomal dominant and autosomal recessive transmission have been demonstrated. In patients of SED tarda, the development up to 5–10 years of age is usually normal after which mild disproportionate trunk and shortening becomes evident. In some patients, the condition remains unrecognized until adolescent years and may become clinically apparent with hip pain, scoliosis or lordosis. SED is rare with its prevalence being approximately 3-4 per million population [3]. The term microdontia (microdentism, microdontism) is defined as the condition of having abnormally small teeth [4]. In generalized microdontia, the teeth are small, the crowns and roots are short, and normal contact areas between the teeth are frequently missing [5]. Shafer, Hine, and Levy divided microdontia into three types: (1) microdontia involving only a single tooth; (2) relative generalized microdontia due to relatively small teeth in large jaws; and (3) true generalized microdontia, in which all the teeth are smaller than normal [6]. According to these authors, aside from its occurrence in some cases of pituitary dwarfism, true generalized microdontia is exceedingly rare. Literature review of MEDLINE/PubMed base did not show any case of SED with microdontia. Here, we report a rare case of SED associated with true generalized microdontia.

## 2. Case Report

**Figure 1:**
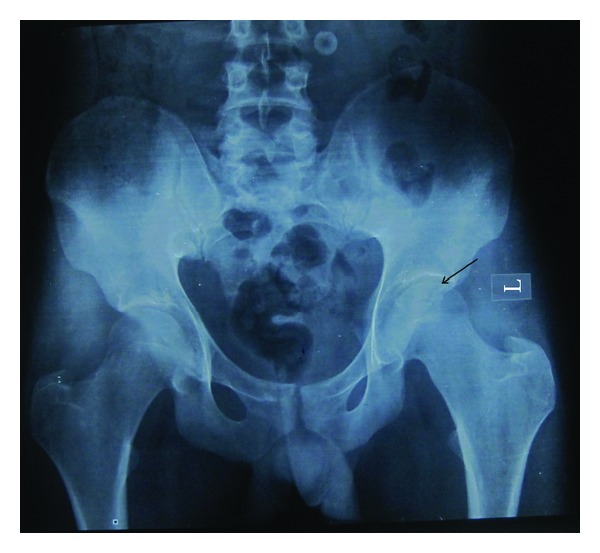
Pelvic X-ray showing spina bifida oculta at L5, femoral epiphysis compressed on left sides.

**Figure 2:**
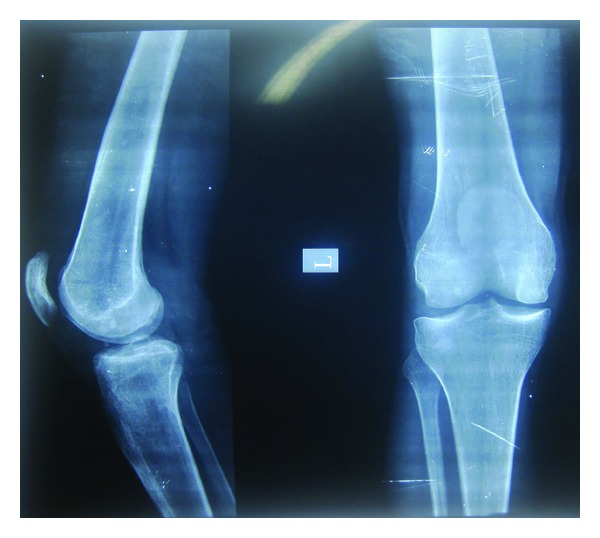
X-ray knee joint showing maldeveloped patella with osteoporosis in right side inferior pubic ramus, lower end of femur, and upper end of tibia.

**Figure 3:**
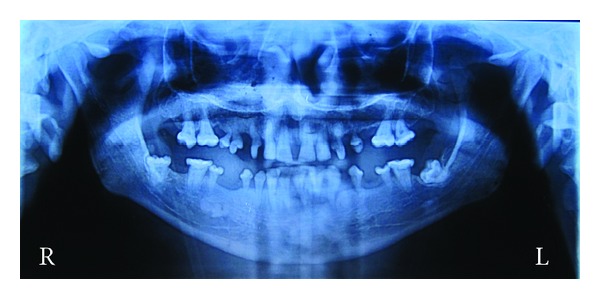
OPG shows conical tooth roots floating over alveolar bone with dilacerations.

A 26-year-old male patient reported with complaint of mobile and missing teeth accompanied with sensitivity to hot and cold since last few months. Patient was not able to eat and chew properly. History of pain in the leg bones while sitting and walking and pain in hand and feet on their movement were present for the last four years. The patient did not complain of pain in any of the joints. Patient also had impaired vision for which he was diagnosed having cataract four years back and was surgically treated for the same then. Patient gave history of hearing deficiency and back pain for past 3 years. On physical examination his height was found to be 65.95 inches with thin stature. Upper segment to lower segment ratio was 0.87, normal being 0.89 to 0.95. this was suggestive of skeletal dysplasia. Hand, wrist, pelvic, and spinal radiographs of the patient were taken. Spinal radiograph revealed loose bodies. Hand wrist radiographs showed normal bone. Pelvic radiographs showed spina bifida oculta at L5 level and femoral epiphysis compressed on left side (Figure 1). Osteoporosis was seen in the inferior pubic ramus right side, lower end of femur, and upper end of tibia. Patella was maldeveloped (Figure 2). DEXA (dual-emission X-ray absorptiometry) scan of the patient was done. DEXA scan score for the patient was between −1 and −2.5. It revealed that patient was osteopenic. Patient was advised calcium supplements and physiotherapy. On oral examination mouth opening was normal. Patient presented with true generalized microdontia. Morphology of the teeth was maintained. Teeth number 23 and 25 were congenitally missing. Caries was present in multiple teeth. Orthopantomogram of the patient showed severe bone loss in relation to all teeth. Teeth number 13, 14, 15, 25, 26, 27, 31, 33, 34, 42, and 44 had dilacerated roots. 37 and 47 were impacted. Teeth appeared small conical floating over alveolar bone. (Figure 3). 44, 34, and 35 were extracted due to extreme mobility and 36 was extracted due to caries. Tooth measurements were made on the diagnostic casts of patient's dentition. Patient was the second of three siblings (2 brothers and one sister); all others did not have any of these findings. There was no family history of similar complaints. Comparison of the patient's tooth measurement was done with anatomic average dental measurements in Tables 1 and 2.

Pedigree and genetic testing showed the condition as autosomal recessive. Based on all the clinical, radiolographical, and genetic findings, final diagnosis of spondyloepiphyseal dysplasia tarda with generalized microdontia was made. Carious teeth were restored with composite restoration and missing teeth were replaced by interim removable partial denture. Physiotherapy is advised to the patient and he is under routine followups.

## 3. Discussion

Spondyloepiphyseal dysplasias are a spectrum of disorders comprising of SED congenita, pseudoachondroplastic SED, and SED tarda [7]. SED congenita is a specific skeletal dysplasia inherited as autosomal dominant disorder which is evident at birth. Cleft palate is common and over half of the patients have high grade myopia and/or retinal detachment. SED tarda is customarily used for the X-linked recessive disorder; however, autosomal dominant and recessive forms have been described. The most consistent radiologic findings in SED are a dysplastic odontoid process, flattened vertebrae, and small and deformed (femoral) epiphyses (long-bone metaphyseal involvement is variable). In the cervical spine of children with SED congenita, atlantoaxial instability is the most commonly encountered and most dangerous problem, found in 30–40%. The major clinical characteristics of SED tarda are pain, stiffness, and limitations to the movements of the lumbar spine and multiple joints combined with a waddling gait as was seen in present case. Progressive symptomatic osteoarthritis of the hips and knees may be seen. Atlantoaxial instability may be present and patients may present with neurologic deficits. Scoliosis or thoracic kyphosis with exaggerated lumbar pain in the back and hips with limitation of motion in these joints are frequent by the teens [2]. Adult height of the patients with SED varies from 52 to 62 inches. In general as a rule, patients with disproportionate stature have some form of skeletal dysplasias, whereas those with relatively normal body proportions have some form of endocrine, nutritional, metabolic, or other nonskeletal defects [8]. Anomaly with ocular, skeletal, craniofacial, dental and somatic developmental defects, termed as Axenfeld-Rieger syndrome (ARS), has been reported [9]. Presentation of the symptoms of the present case were quite different than that of ARS except for the hypodontia and the microdontia. In ARS microdontia is generally found in the bimaxillary anterior region, whereas in our case microdontia was generalized. Besides from its occurrence in some cases of pituitary dwarfism, true generalized microdontia is exceedingly rare [10]. Cleft palate and missing teeth are frequently reported to be associated with SED. True generalized microdontia associated with SED has never been reported in literature. The patient had two missing teeth but no cleft was present. The initiating factor or factors responsible for microdontia remain obscure. Mutation in developmental regularity genes is known to cause variety of dental defects [11]. Both genetic and environmental factors are involved in the complex etiology of microdontia The development of a tooth has been shown to have ectodermal, mesodermal, and neural crest contributions. The variation in size of a particular tooth arises during the period when the form of the tooth is being determined by the enamel organ and the sheath of Hertwig at the bell stage of enamel organ. The determination of the form of the crown is thought to be related to different regions of the oral epithelium or to the ectomesenchyme. Studies have shown that different regions of the oral epithelium rather than the underlying ectomesenchyme are initially responsible for the shape of the crown [12] Bones dating from the middle ages which were excavated at Alborg, Denmark, proved evidence for generalized microdontia resulting from intrauterine growth retardation [13]. There is no cure for spondyloepiphyseal dysplasia, but a great deal can be done to provide support and compensate for disabilities. An ophthalmologist should examine the patient at an early stage. Congenital cataracts should be treated surgically. Recurrent middle ear infections should be treated to prevent hearing impairment. Hip joint defects and arthrosis sometimes require surgical intervention. Spinal defects are treated with a corset brace but may require surgery. Microdonts having good root length and bone support are given crowns. If microdonts are lost prematurely, they can then be replaced by removable partial dentures. In adults dental implants can be used with normal-sized teeth. We have presented a case of SED with generalized microdontia here and future studies are needed to find the association basis between the symptoms and there prevalence.

## Figures and Tables

**Table 1:** Measurements in millimeters were made on clinical crown of diagnostic casts (right side).

Tooth (right side) (U/L)	Anatomic average crown length of teeth (U/L)	Crown length of patient's teeth (U/L)	Anatomic average mesiodistal width of teeth (U/L)	Mesiodistal width of patient's teeth (U/L)
Central incisor	10.5/9	6.5/5	8.5/5	6/4.5
Lateral incisor	9/9.5	5/4	6.5/55	5/5
Canine	10/11	6/5.5	7.5/7	5/4
First premolar	8.5/8.5	5/—	7/7	5/—
Second molar	8.5/8.5	3.5/—	7/7	3.5/—
First molar	7.5/7.5	4.5/—	10/11	8/—
Second molar	7/7.5	5/—	9/10.5	7.5/—
Third molar	6.5/7	—	8.5/9.5	—

Anatomic averages taken from *Textbook of Dental Anatomy and Physiology, R. C. Wheeler*.

**Table 2:** Measurements in millimeters were made on clinical crown of diagnostic casts (left side).

Tooth (left side) (U/L)	Anatomic average crown length of teeth (U/L)	Crown length of patient's teeth (U/L)	Anatomic average mesiodistal width of teeth (U/L)	Mesiodistal width of patient's teeth (U/L)
Central incisor	10.5/9	5/5.5	8.5/5	7/4.5
Lateral incisor	9/9.5	5/5.5	6.5/55	6/4
Canine	10/11	5.5/5.5	7.5/7	5/5
First premolar	8.5/8.5	4.5/5	7/7	4.5/5
Second molar	8.5/8.5	—/2	7/7	—/5
First molar	7.5/7.5	4/4	10/11	8/3.5
Second molar	7/7.5	—	9/10.5	6/—
Third molar	6.5/7	—	8.5/9.5	—

Anatomic averages taken from *Textbook of Dental Anatomy and Physiology, R. C. Wheeler*.
